# Perioperative knowledge needs in maxillofacial trauma patients: a cross-sectional survey

**DOI:** 10.1186/s12903-025-07378-0

**Published:** 2026-01-16

**Authors:** QingYan Xiong, XiaoRong Zhou, WenYu Lai, YiXin Liu, Jie Lin, YongLe Shi

**Affiliations:** 1 West China School of Nursing, Sichuan University, Chengdu, Sichuan China; 2 National Center for Stomatology & National Clinical Research Center for Oral Diseases & Department of Emergency, West China Hospital of Stomatology, Sichuan University, Chengdu, Sichuan 610041 China; 3 State Key Laboratory of Oral Diseases & National Center for Stomatology & National Clinical Research Center for Oral Diseases, West China Hospital of Stomatology, Sichuan University, Chengdu, China

**Keywords:** Craniofacial trauma, Craniofacial soft tissue injury, Health knowledge need, Health education

## Abstract

**Background:**

Maxillofacial trauma poses a growing public health burden worldwide, yet the perioperative knowledge needs of affected patients are poorly understood across different demographic groups. This study aimed to evaluate the perioperative knowledge needs in patients with maxillofacial trauma across different demographic groups, while re-evaluating the reliability and validity of the research instrument.

**Method:**

A cross-sectional survey was conducted among 469 maxillofacial trauma patients at a tertiary stomatology hospital in Sichuan, using convenience sampling. Structural validity of the questionnaire was assessed via exploratory factor analysis (EFA), while reliability was evaluated through internal consistency (Cronbach’s alpha) and split-half reliability. Non-parametric tests (Kruskal-Wallis H and Mann-Whitney U) were employed for inferential analysis.

**Results:**

The cohort comprised 64.2% (301/469) male participants, 77.4% (363/469) of whom were aged 16 years or younger. EFA revealed a six-dimensional structure that explained 69% of the variance. The questionnaire demonstrated strong reliability (Cronbach’s alpha = 0.889, split-half = 0.780). Significant variations in knowledge needs were observed across the perioperative stages (*p* < 0.001). Age significantly influenced needs relating to routine postoperative care (*p* < 0.001), medications/secondary care (*p* < 0.005), and scar prevention/treatment (*p* < 0.001).

**Conclusion:**

The knowledge needs of patients vary significantly according to their demographics, peaking during the first week after surgery. This highlights the importance of personalized patient education during the early stages of recovery.

**Trial registration:**

Clinical Trial Registration No. ChiCTR2300079287, Registration Date 29 December 2023.

## Introduction

 Maxillofacial trauma represents a substantial global public health challenge, constituting the most prevalent form of trauma encountered in dental emergency settings. Epidemiologic data indicate these injuries account for nearly 50% of the 12 million annual trauma admissions to U.S. emergency departments, representing 7–10% of total emergency department visits, with comparable incidence rates observed in China [1–3]. Primary etiological factors demonstrate demographic and geographic variations, encompassing motor vehicle accidents, falls/collisions (involving impact with floors, walls, stairs, or furniture), interpersonal violence, and sports-related injuries [4–6]. Current clinical protocols primarily involve debridement and suturing as first-line interventions, with retrospective cohort studies reporting 71.5% of patients receiving this treatment modality [3]. Inadequate postoperative care may precipitate perioperative complications, including wound infection, impaired healing, and pathological scarring. Inadequate patient understanding of complication risks and limited self-efficacy may decrease care adherence. Strengthening health beliefs can increase the perceived value of postoperative care, leading to better health outcomes [7]. While generally non-life-threatening compared to visceral or neurovascular trauma, maxillofacial injuries present distinct perioperative management challenges due to the anatomical region’s dual functional and aesthetic significance. This complex interface supports essential physiological functions (mastication, articulation, respiration) and social communication through facial expressivity. Post-traumatic sequelae, particularly disfiguring scars, are associated with long-term functional impairments and psychosocial morbidity [8–10]. The results of the study showed a variety of outcomes ranging from body image dissatisfaction and social isolation to clinically diagnosed depression [11, 12]. Perioperative knowledge encompasses the skills and understanding required throughout the entire surgical process, from the preoperative to the postoperative stages. Understanding this knowledge can encourage patient cooperation with medical care and reduce anxiety to some extent [13, 14]. Given that postoperative care primarily occurs in home settings through patient self-management, understanding perioperative knowledge requirements is critical for optimizing self-care efficacy and wound prognosis. Despite the clinical importance of debridement and suturing in the treatment of maxillofacial trauma, the existing literature has predominantly focused on epidemiological studies and the factors influencing wound healing [1–3, 8, 15]. Limited research has been conducted on the knowledge needs of patients following debridement and suturing for maxillofacial trauma. Building upon our research team’s prior investigations [16], this study aims to systematically assess perioperative knowledge requirements in this patient population. The findings are anticipated to inform targeted nursing education strategies, ultimately enhancing perioperative management quality and healthcare satisfaction. To ensure methodological rigor, we recalibrated the survey instrument through dimensional restructuring and conducted a comprehensive reliability reassessment following adjustments to the target sample population.

## Material & methods

### Study design and population

All participants provided written informed consent. It was a cross-sectional study conducted at the third-class hospital of stomatology in Sichuan province between January 2025 to May 2025. The inclusion criteria for survey participants were as follows, patients with maxillofacial trauma or their caregivers (In this study, patients under the age of 16 or with cognitive impairment were represented by their caregivers.), undergoing debridement and suturing, follow-up after debridement and suturing, basic communication and reading skills, willingness to participate in this study, and vegetable informed consent. Exclusion Criteria included individuals with mid- or late-stage withdrawals, and comorbid serious organic pathologies. A sample size of 10 participants per scale item was targeted, consistent with best practices in measurement tool validation. The scale of this study consisted of 20 items, and the minimum sample size required was 200. Considering 10% invalid questionnaires, the final sample size was 223.

### Data collection

The general information questionnaire included the patient’s perioperative stage (POS), age, gender, and site of injury. Data was collected by researchers through face-to-face interviews with patients or their caregivers. The Knowledge Needs Scale [16] was self-designed by our research team in advance by reviewing the literature, surveying patients, and consulting with experts, formulated in Chinese, and included 20 items in 4 dimensions to assess the knowledge needs of primary caregivers of patients after maxillofacial debridement and suturing for home care. All questions were scored on a Likert 5-point scale (1 = very little need, 5 = very much need), with a total score of 20 to 100, with higher scores indicating a higher level of need for home care knowledge among primary caregivers of patients after maxillofacial trauma debridement and suturing. Seven experts who had worked in the professional field of stomatognathic surgery for 5 years or more, with intermediate or senior titles, and with undergraduate degrees or higher were invited to participate in the expert seminar (among them, 4 experts in stomatognathic medicine and 3 experts in nursing), to discuss and modify the content, structure, and the logical relationship between the entries of the questionnaire, The overall Cronbach’s alpha for the scale was 0.952 and the split-half reliability was 0.835, both of which were higher than 0.70, and The Cronbach’s alpha for the dimensions ranged from 0.860 to 0.960, with split-half reliabilities ranging from 0.743 to 0.948. *The scale content is shown in* Table 1.

**Table 1:** Perioperative knowledge needs scale for maxillofacial trauma patients

Dimension	Items
Knowledge of routine wound care	Suture material knowledge
	Suture removal knowledge
	Wound dressing knowledge
	Wound irrigation/disinfection
	Postoperative daily life care
. Wound complication prevention and treatment	Wound infection prevention and treatment
	Wound bleeding prevention and treatment
	Wound dehiscence prevention and treatment
	Scab-related management
Medications and secondary care	Late-stage fracture treatment
	Immunoglobulin/vaccine use knowledge
	Antibiotic use knowledge
Scar Management and Other Procedures	Postoperative diet and nutrition guidance
	Postoperative activity guidance
	Postoperative pain management
	Pharmacological scar treatment
	Non-pharmacological scar treatment
	Prevention of maxillofacial trauma recurrence
	Emergency home care of recurrent trauma
	Healing characteristics of recurrent trauma

### Statistical analyses

SPSS 27.0 software was used for data entry and analysis. The structural validity of the questionnaire was evaluated using exploratory factor analysis (EFA), while the reliability of the questionnaire was assessed through internal consistency reliability (Cronbach’s alpha) and split-half reliability. The quantitative data were tested for normality, and non-normally distributed quantitative data were described using the median and interquartile range [M(IQR)]. Non-parametric tests (Kruskal-Wallis H and Mann-Whitney U) were employed for inferential analysis. All statistical tests were two-sided, with the significance level set at α = 0.05. Differences were deemed statistically significant at *p* < 0.05.

## Results

### Demographic data

A total of 469 questionnaires were collected in this study. There were 168 females (35.8%) and 301 males (64.2%), with 58.8% aged 0–7 years old. The most commonly trauma area was the forehead, accounting for 24.5%. See Table 2 for more details.

**Table 2:** Demographic data(*N* = 469)

Category	*n*(%)
*Age in years*	
0–7	276(58.80)
8–16	87(18.60)
17–49	94(20.00)
>50	12(2.60)
*Gender*	
Female	168(35.80)
Male	301(64.20)
*Postoperative days*	
POS 0	118(25.20)
POS1-5	180(38.40)
POS 6–10	158(33.70)
POS ≥ 11	13(2.80)
*Location of injury*	
Scalp	3(0.60)
Forehead	115(24.50)
Orbit region	38(8.10)
Supercilium	25(5.30)
Temporal	1(0.20)
Cheek	35(7.50)
Front of the ear	4(0.90)
Nose	9(1.90)
Upper lip	89(19.00)
Lower lip	77(16.40)
Chin	51(10.90)
Intra-oral	22(4.70)

### Reliability and validity analysis

**Fig. 1:**
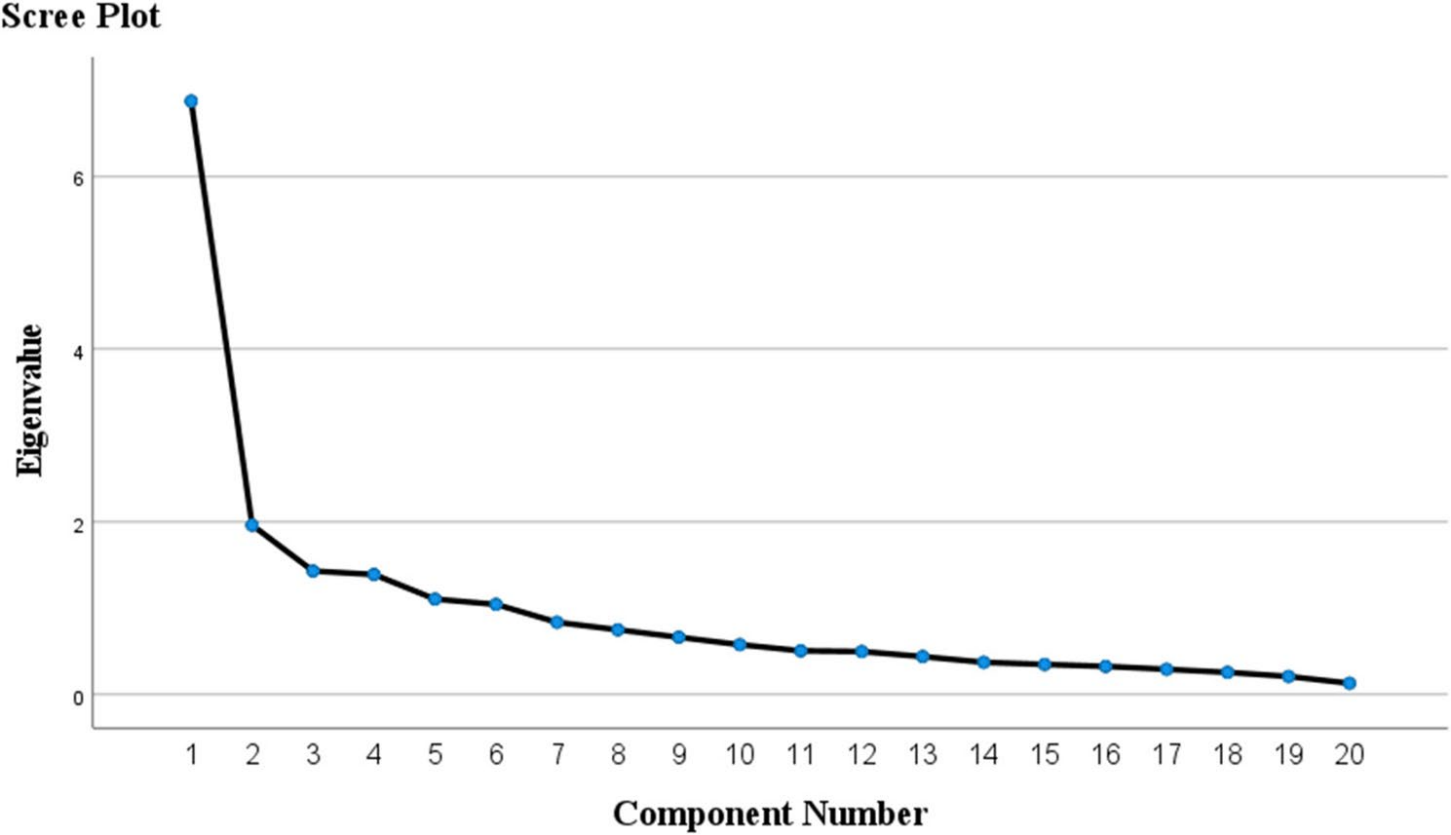
Scree plot of eigenvalues for principal component analysis

The Kaiser-Meyer-Olkin (KMO) measure of sampling adequacy was 0.861, and Bartlett’s test of sphericity yielded a significant χ² value of 4419.222 (*p* < 0.001), confirming the suitability of the data for factor analysis. EFA revealed a six-factor solution. The scree plot indicated a six-factor solution (Fig. 1), collectively accounting for 69.0% of the total variance (Table 3). Based on item content, the dimensions were labeled as follows: Knowledge of routine wound care, Wound complication prevention and treatment, Medications and secondary care, Routine postoperative care, Scar prevention and treatment, Maxillofacial trauma prevention and treatment (Tables 4 and 5).

**Table 3:** Total variance explained by principal components

Component	Initial Eigenvalues			Rotation Sums of Squared Loadings		
	Total	% Variance	Cumulative %	Total	% Variance	Cumulative %
1	6.87	34.39	34.39	2.80	14.02	14.02
2	1.96	9.81	44.20	2.60	13.03	27.06
3	1.43	7.14	51.35	2.54	12.74	39.80
4	1.39	6.95	58.30	2.00	10.03	49.84
5	1.10	5.52	63.82	1.96	9.80	59.65
6	1.04	5.21	69.04	1.87	9.39	69.04
7–20	< 1.00	< 5.00	-	-	-	-

**Table 4:** Rotated component matrix (Varimax Rotation)

Item	1	2	3	4	5	6
5. Wound infection prevention and treatment	0.85					
7. Wound dehiscence prevention and treatment	0.82					
6. Wound bleeding prevention and treatment	0.79					
8. Wound scab-related management	0.50					
13. Postoperative daily life care		0.81				
14. Postoperative activity guidance		0.76				
12. Postoperative diet and nutrition guidance		0.66				
15. Postoperative pain management		0.53				
18. Prevention of maxillofacial trauma recurrence			0.83			
19. Emergency home care of recurrent trauma			0.78			
20. Healing characteristics of recurrent trauma			0.77			
2. Suture removal knowledge				0.76		
1. Suture material knowledge				0.71		
3. Wound dressing knowledge				0.55		
4. Wound irrigation/disinfection knowledge				0.52		
17. Non-pharmacological scar treatment					0.91	
16. Pharmacological scar treatment					0.90	
10. Immunoglobulin/vaccine use knowledge						0.76
9. Late-stage fracture treatment						0.64
11. Antibiotic use knowledge						0.61

**Table 5:** Perioperative knowledge needs scale for maxillofacial trauma patients

Dimension	Items
Knowledge of routine wound care	Suture material knowledge
	Suture removal knowledge
	Wound dressing knowledge
	Wound irrigation/disinfection
. Wound complication prevention and treatment	Wound infection prevention and treatment
	Wound bleeding prevention and treatment
	Wound dehiscence prevention and treatment
	Scab-related management
Medications and secondary care	Late-stage fracture treatment
	Immunoglobulin/vaccine use knowledge
	Antibiotic use knowledge
Routine postoperative care	Postoperative daily life care
	Postoperative diet and nutrition guidance
	Postoperative activity guidance
	Postoperative pain management
Scar prevention and treatment	Pharmacological scar treatment
	Non-pharmacological scar treatment
Maxillofacial trauma prevention and treatment	Prevention of maxillofacial trauma recurrence
	Emergency home care of recurrent trauma
	Healing characteristics of recurrent trauma

Reliability analysis demonstrated strong internal consistency, with an overall Cronbach’s α of 0.889 and split-half reliability of 0.780 (both exceeding the threshold of 0.7) (Table 6). The Cronbach’s α values for individual dimensions ranged from 0.881 to 0.892, while split-half reliability ranged from 0.69 to 0.92 (Table 6). The perioperative knowledge needs of oral and maxillofacial trauma patients are detailed in Table 5.

**Table 6:** Cronbach’s α and Guttman Split-Half coefficient

Dimension	Cronbach’s α	Split-Half Coefficient
Total	0.88	0.78
Knowledge of routine wound care	0.76	0.69
Wound complication prevention and treatment	0.76	0.81
Medications and secondary care	0.75	0.71
Routine postoperative care	0.74	0.85
Scar prevention and treatment	0.79	0.92
Maxillofacial trauma prevention and treatment	0.75	0.83

### Knowledge needs score

The Kolmogorov-Smirnov (K-S) test revealed that the scores of all 20 items and the four dimensions in maxillofacial trauma patients or their caregivers significantly deviated from a normal distribution (D = 0.130–0.493, *p* < 0.001). Consequently, the data were described using medians and interquartile ranges (IQRs). As presented in Table 7.

**Table 7:** Average, median, and interquartile range for dimensions of perioperative knowledge needs score

Dimension	M	*M* (P25, P75)
Knowledge of routine wound care	4.75	4.75(4.25, 5.00)
Wound complication prevention and treatment	5.00	4.50(4.50, 5.00)
Medications and secondary care	4.00	4.00(3.67, 4.67)
Routine postoperative care	5.00	4.50(4.50, 5.00)
Scar prevention and treatment	5.00	5.00(5.00, 5.00)
Maxillofacial trauma prevention and treatment	4.67	4.67(4.33, 5.00)

### Current status of perioperative knowledge needs for maxillofacial trauma

**Fig. 2:**
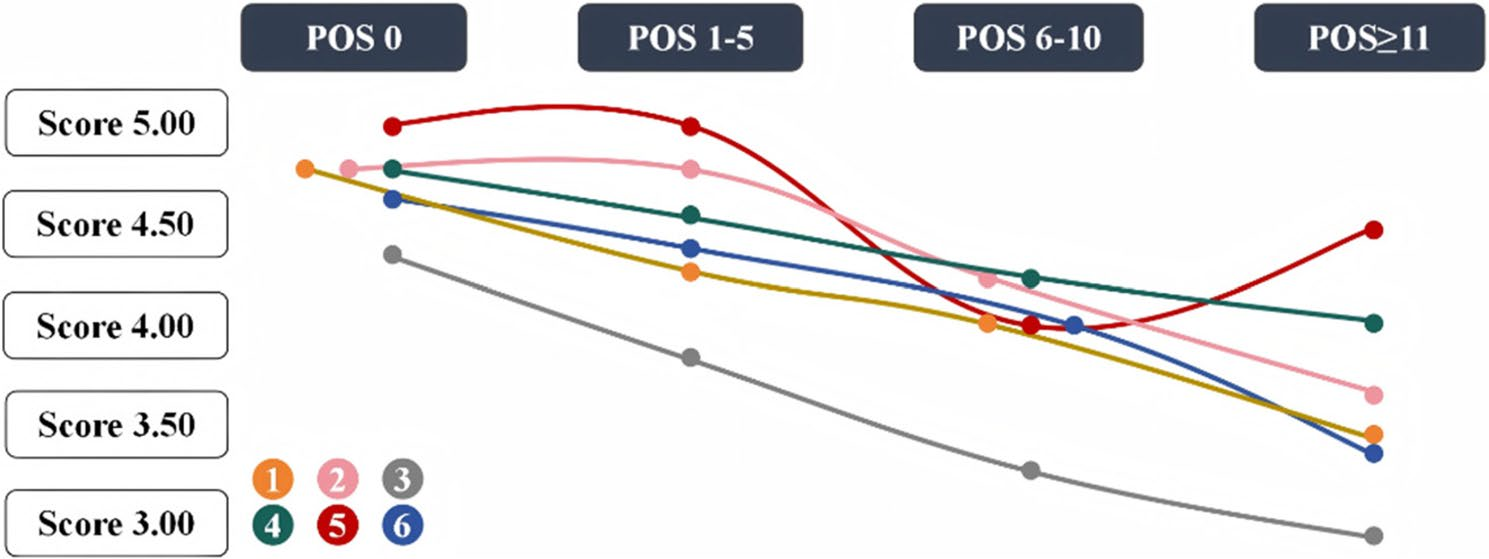
Change in Perioperative Patient Knowledge Needs. Figure legend: 1. Knowledge of routine wound care；2. Wound complication prevention and treatment；3.Medications and secondary care；4.Routine postoperative care；5.Scar prevention and treatment；6.Maxillofacial trauma prevention and treatment; POS (patient's perioperative stage)

The results of the analysis showed a statistically significant impact of perioperative stage on patient knowledge needs in all of the following measured domains: routine wound care, prevention and management of postoperative wound complications, medications and secondary care, routine postoperative care, scar prevention and management, and maxillofacial trauma prevention and treatment (η²=0.034–0.131, *p* < 0.001 for all comparisons) (Table 8; Fig. 2). Age was likewise significantly associated with knowledge needs in routine postoperative care and maxillofacial trauma prevention/treatment (η²=0.037༆0.043, *p* < 0.001) (Table 9). There was also a significant difference in knowledge needs regarding scar prevention and treatment (η²=0.077, *p* < 0.001) (Table 10). However, the differences were not significant at the gender level (Table 11).

**Table 8:** Educational needs in maxillofacial trauma patients by perioperative stage (*N* = 469)

Dimension	POS 0	POS 1–5	POS 6–10	POS ≥ 11	H	*p*	η²
	M (P25, P75)						
1	5.00(4.75,5.00)	4.75(4.25,5.00)	4.50(4.00,5.00)	4.75(3.50,5.00)	62.16	< 0.001	0.130
2	5.00(4.75,5.00)	5.00(4.75,5.00)	4.75(4.25, 5.00)	4.50(3.60,5.00)	37.57	< 0.001	0.076
3	4.50(4.33,5.00)	4.00(3.66,4.33)	3.66(3.33, 4.33)	3.33(2.80,4.30)	62.79	< 0.001	0.131
4	5.00(4.75,5.00)	5.00(4.50,5.00)	4.75(4.25,5.00)	4.75(4.00,5.00)	26.66	< 0.001	0.052
5	5.00(5.00,5.00)	5.00(5.00,5.00)	5.00(4.00,5.00)	5.00(4.50,5.00)	24.25	< 0.001	0.047
6	5.00(4.66,5.00)	4.66(4.33,5.00)	4.66(4.00,5.00)	5.00(3.30,5.00)	18.42	< 0.001	0.034

1. Knowledge of routine wound care;2. Wound complication prevention and treatment༛3.Medications and secondary care༛4.Routine postoperative care༛5.Scar prevention and treatment༛6.Maxillofacial trauma prevention and treatment; POS (patient’s perioperative stage)

**Table 9:** Educational needs in maxillofacial trauma patients by age group (*N* = 469)

Dimension	0–7	8–16	17–49	≥ 50	H	*p*	η²
	M (P25, P75)						
1	5.00(4.50, 5.00)	5.00(4.25, 5.00)	4.75(4.18, 5.00)	4.50(4.00, 4.75)	7.56	0.056	0.010
2	5.00(4.50, 5.00)	5.00(4.50, 5.00)	4.75(4.50, 5.00)	4.75(4.13, 5.00)	2.99	0.3932	0.000
3	4.33(3.67, 5.00)	4.33(3.33, 5.00)	3.66(3.33, 4.33)	4.16(3.08, 4.33)	13.91	0.003	0.024
4	5.00(4.50, 5.00)	5.00(4.50, 5.00)	4.50(4.00, 5.00)	4.50(3.81, 5.00)	19.84	< 0.001	0.037
5	5.00(5.00, 5.00)	5.00(5.00, 5.00)	5.00(5.00, 5.00)	4.00(3.25, 5.00)	13.03	0.005	0.022
6	5.00(4.33, 5.00)	5.00(4.33, 5.00)	4.33(4.00, 5.00)	4.17(3.75, 5.00)	22.42	< 0.001	0.043

1. Knowledge of routine wound care;2. Wound complication prevention and treatment༛3.Medications and secondary care༛4.Routine postoperative care༛5.Scar prevention and treatment༛6.Maxillofacial trauma prevention and treatment

**Table 10:** Educational needs in maxillofacial trauma patients by site of injury (*N* = 469)

Dimension	1	2	3	4	5	6
	M (P25, P75)					
Scalp	5.00(NA)	5.00(NA)	4.33(NA)	5.00(NA)	5.00(NA)	5.00(NA)
Forehead	4.75(4.25, 5.00)	5.00(4.50, 5.00)	4.00(3.66, 4.66)	5.00(4.50, 5.00)	5.00(5.00, 5.00)	4.66(4.33, 5.00)
Orbit region	5.00(4.43, 5.00)	5.00(4.43, 5.00)	4.00(3.66, 5.00)	5.00(4.43, 5.00)	5.00(5.00, 5.00)	4.66(4.33, 5.00)
Supercilium	4.75(4.50, 5.00)	5.00(4.50, 5.00)	3.50(3.66, 5.00)	5.00(4.37, 5.00)	5.00(5.00, 5.00)	5.00(4.00, 5.00)
Temporal	NA	NA	NA	NA	NA	NA
Cheek	4.75(4.25, 5.00)	5.00(4.75, 5.00)	4(3.65, 4.33)	5.00(4.50, 5.00)	5.00(5.00, 5.00)	4.66(4.00, 5.00)
Front of the ear	4.62(4.12, 4.93)	4.87(4.37, 5.00)	3.33(2.75,3.91)	4.75(4.50, 5.00)	4.50(4.00, 5.00)	4.83(4.41, 5.00)
Nose	4.75(4.25, 4.75)	5.00(4.75, 5.00)	4.33(3.16, 5.00)	4.62(4.50, 5.00)	5.00(5.00, 5.00)	4.66(4.33, 5.00)
Upper lip	5.00(4.25, 5.00)	5.00(4.50, 5.00)	4.00(3.33 5.00)	4.75(4.25, 5.00)	5.00(4.25, 5.00)	5.00(4.33, 5.00)
Lower lip	4.75(4.25, 5.00)	5.00(4.50, 5.00)	4.33(3.33, 5.00)	5.00(4.50, 5.00)	5.00(5.00, 5.00)	4.66(4.00, 5.00)
Chin	5.00(4.50, 5.00)	5.00(4.50, 5.00)	4.33(3.66, 4.66)	5.00(4.50, 5.00)	5.00(5.00, 5.00)	5.00(4.33, 5.00)
Intra-oral	4.87(4.25, 5.00)	4.50(4.25, 5.00)	4.00(3.58, 4.33)	5.00(4.50, 5.00)	3.50(3.00, 5.00)	4.66(4.25, 5.00)
H	7.84	11.65	6.91	5.8	45.22	6.19
P	0.727	0.39	0.806	0.886	< 0.001	0.86
η²	0.000	0.002	0.000	0.000	0.077	0.000

1. Knowledge of routine wound care;2. Wound complication prevention and treatment༛3.Medications and secondary care༛4.Routine postoperative care༛5.Scar prevention and treatment༛6.Prevention and treatment of maxillofacial trauma; NA (Not applicable)

**Table 11:** Educational needs in maxillofacial trauma patients by Gender(*N* = 469)

Dimension	Male	Female	Z	*p*	*r*
	M (P25, P75)				
1	4.75(4.25, 5.00)	5(4.25, 5.00)	−0.66	0.508	0.031
2	5(4.50, 5.00)	5(4.50, 5.00)	−0.39	0.69	0.018
3	4(3.67, 5.00)	4(3.66, 4.66)	−1.08	0.278	0.050
4	5(4.50, 5.00)	5(4.50, 5.00)	−0.16	0.872	0.008
5	5(5.00, 5.00)	5(5.00, 5.00)	−0.34	0.728	0.016
6	5(4.33, 5.00)	4.66(4.33, 5.00)	−0.01	0.987	0.001

1. Knowledge of routine wound care;2. Wound complication prevention and treatment༛3.Medications and secondary care༛4.Routine postoperative care༛5.Scar prevention and treatment༛6.Maxillofacial trauma prevention and treatment

## Discussion

The research instrument used in this study was the team’s previous research, and because maxillofacial trauma in children accounts for 70%−80% of the total maxillofacial trauma volume [3, 17, 18], the original scale’s target measurement population was the primary caregivers of children who underwent maxillofacial debridement and suture closure. The reliability and validity of the scale were good. However, in the follow-up clinical work, we found that middle-aged and elderly people accounted for a significant proportion of maxillofacial trauma, so the present study did not restrict the age of the included population, and the original tool’s entries were adjusted. The modified questionnaire was tested for reliability and validity, and the statistical results showed that the items we added did not contribute to the scale as a whole and were therefore excluded, with a Cronbach’s α of 0.889 and a split-half reliability of 0.780, both of which were >0.7. An exploratory factor analysis was used to explore the dimensions included in the pool of items as well as the structure of the scale, which was finally divided into six dimensions, which were as follows: Knowledge of routine wound care, Prevention and treatment of wound complications, Medications and secondary care, Routine postoperative care, Scar prevention and treatment, Prevention and treatment of maxillofacial trauma.

The cross-sectional survey revealed significant differences in perioperative knowledge needs among patients or their caregivers with maxillofacial trauma across different surgical stages (*p* < 0.001). Specifically, the progressive decline in knowledge needs scores observed across Dimensions 1 to 4 and Dimension 6 over time can be scientifically explained through the physiological mechanisms of wound healing. Current research has established that wound healing represents a precisely orchestrated biological process, characterized by four distinct yet overlapping phases: hemostasis, inflammation, proliferation, and remodeling [19, 20]. This complex process involves the synergistic interaction of multiple biological components, including inflammatory cells, cytokines, repair cells, extracellular matrix components, and various molecular mediators, which collectively establish a sophisticated repair mechanism [21]. Of particular significance is the initial 72-hour period post-injury, during which the body undergoes two critical phases hemostasis and the inflammatory response. Clinical observations indicate that peak wound exudation typically occurs between 24 and 48 h representing a critical therapeutic window for effective wound management [19–21]. This temporal pattern aligns with our findings that patients’ perioperative needs are predominantly concentrated in the early postoperative period, emphasizing the importance of achieving wound closure during this initial phase to facilitate efficient healing. To ensure favorable healing outcomes, the implementation of a systematic and comprehensive perioperative wound assessment protocol is essential. This approach should incorporate several key components: (1) selection of appropriate wound cleansing techniques tailored to individual patient needs; (2) customization of functional dressings based on wound characteristics; and (3) implementation of evidence-based pain management strategies. These elements directly align with the content of Dimensions 1–4 of our knowledge needs scale, which comprehensively address critical aspects, including wound, dressing selection, pain management, and early postoperative care. Due to the rich vascular supply of facial tissues, sutures in maxillofacial injuries are typically removed within 5–10 days [22]. However, if wound healing progresses well, clinicians may consider early, intermittent suture removal as early as 5 days post-surgery [22]. The demand for postoperative wound care information follows a distinct temporal pattern, consistent with the perceived severity construct of the Health Belief Model [23]. In the immediate postoperative phase (days 05), when wound trauma is most pronounced, patients and caregivers frequently overestimate risk severity due to visible symptoms. This period is marked by heightened information-seeking behavior, as individuals seek professional guidance to mitigate excessive concerns—such as fears about suture failure or complications. Notably, as patient and caregiver experience in wound management accumulates, their reliance on informational support tends to decrease. This trend highlights the importance of targeted health education by healthcare professionals during the critical perioperative phase, where knowledge needs are most pronounced. Dimension 5 exhibited significantly higher scores than other dimensions during the immediate postoperative period (0–5 days) and 11 days post-surgery. A transient decline in scores was observed between postoperative days 6–10, followed by a subsequent recovery to baseline levels by day 11. This phenomenon may be attributed to the unique anatomical and psychosocial significance of maxillofacial injuries. As the facial region is integral to essential functions (e.g., mastication, verbal communication) while also serving critical aesthetic and social roles [15], patients or their caregivers demonstrate heightened concern regarding scar formation and repair from the initial injury phase. Consequently, Dimension 5 maintained significantly higher scores than other dimensions. At postoperative day 11, when standard healing is achieved and sutures are removed, patients or their caregivers typically begin scar revision. The selection among various treatment options - including medical therapies, physical treatments (laser, cryotherapy) [24, 25], and surgical approaches - requires patients to understand each method’s specific requirements and applicability. This need for detailed treatment information likely drives the heightened demand for scar revision knowledge observed beyond postoperative day 11.

Age demonstrated statistically significant associations with three dimensions: routine postoperative care (*p* < 0.001), medication and secondary care (*p* < 0.05), and scar prevention and treatment (*p* < 0.001). Patients aged ≤ 16 years scored significantly higher than adults across all three dimensions. Notably, scores in two dimensions (excluding medication and secondary care) exhibited a progressive decline with increasing age. This pattern may reflect several factors: (1) Pediatric patients represent a vulnerable population, prompting primary caregivers to prioritize meticulous wound care to optimize healing outcomes and support normal growth/psychological development [26, 27]; (2) Preschool-aged children face elevated injury risks due to developmental characteristics, including gait instability, poor balance, heightened environmental curiosity, and limited self-protection awareness - all contributing to increased fall susceptibility [28–30]. Our analysis revealed significantly higher scores in the medication and secondary care dimension among participants aged ≥ 50 years than the 17–49 age group (*p* < 0.05). This disparity may be attributed to age-related physiological changes and the increased prevalence of multimorbidity in older populations [31–33]. Epidemiological data indicate that approximately 40% of adults aged ≥ 50 years experience multimorbidity, with this proportion exceeding 50% in those aged ≥ 60 years [33, 34]. The greater disease burden and heightened motivation for optimal postoperative recovery likely contribute to the enhanced interest in postoperative medication management and secondary surgical care knowledge observed in this demographic. Overall, different populations prioritize health behaviors based on their perceptions of health threats, vulnerability, severity, and anticipated benefits [23]. Objective factors, such as multiple health conditions or specific developmental stages, often significantly influence their decision-making.

### Limitations and future research

This study offers valuable insights into personalized care by characterizing variations in patient knowledge needs, yet several limitations warrant attention. First, the findings are specific to patients with maxillofacial trauma undergoing debridement and primary closure at a single institution, potentially limiting generalizability due to cultural and population-specific factors. Future multicenter studies incorporating diverse cultural and geographic populations are needed to enhance external validity. Second, the age distribution of the study population was naturally skewed due to clinical sampling limitations, reflecting disease onset characteristics. While this skew could not be artificially adjusted, extending the survey duration in future studies could achieve a more balanced age distribution, improving the robustness and representativeness of findings. Third, the number of adult participants was relatively insufficient due to clinical sample distribution characteristics. To address this, future research will extend the sample collection period to balance representation across age groups. Additionally, optimizing scales and expanding the study population will further enhance the generalizability of findings.

## Conclusion

This study successfully modified and validated the Home Care Knowledge Needs Scale for Primary Carers of Postoperative Maxillofacial Trauma Patients, creating a reliable and valid tool for assessing postoperative knowledge requirements. Our findings show that caregivers’ knowledge needs are most critical during the early postoperative period, with the highest demand observed in scar prevention and treatment, followed by wound care and medication management. These insights emphasize the need for structured, phase-specific education programs to effectively address knowledge gaps. Use this scale as a routine assessment tool during postoperative follow-ups to identify caregivers’ specific knowledge deficits. Early-stage education interventions should be implemented, particularly during the first two weeks post-surgery when knowledge needs are most acute. Multimodal teaching methods should be adopted to enhance comprehension and adherence, and education content should be personalized based on individual caregiver profiles to maximize learning outcomes.

## Data Availability

The datasets used and/or analyzed during the current study are available from the corresponding author on reasonable request.
