# Correction: Perioperative knowledge needs in maxillofacial trauma patients: a crosssectional survey

**DOI:** 10.1186/s12903-026-08139-3

**Published:** 2026-04-16

**Authors:** QingYan Xiong, XiaoRong Zhou, WenYu Lai, YiXin Liu, Jie Lin, YongLe Shi

**Affiliations:** 1 West China School of Nursing, Sichuan University, Chengdu, Sichuan China; 2 National Center for Stomatology & National Clinical Research Center for Oral Diseases & Department of Emergency, West China Hospital of Stomatology, Sichuan University, Chengdu, 610041 Sichuan China; 3 State Key Laboratory of Oral Diseases & National Center for Stomatology & National Clinical Research Center for Oral Diseases, West China Hospital of Stomatology, Sichuan University, Chengdu, China

**Correction: BMC Oral Health 26, 272, (2026)**

**https://doi.org/10.1186/s12903-025-07378-0**

In this article [1], YongLe Shi should have been denoted as a corresponding author.

The original article has been corrected.
